# C-type lectins, fungi and Th17 responses

**DOI:** 10.1016/j.cytogfr.2010.10.001

**Published:** 2010-12

**Authors:** Simon Vautier, Maria da Glória Sousa, Gordon D. Brown

**Affiliations:** Aberdeen Fungal Group, Section of Infection and Immunity, Institute of Medical Sciences, School of Medicine and Dentistry, University of Aberdeen, Aberdeen AB25 2ZD, UK

**Keywords:** Th17, C-type lectin, Syk kinase, Fungi, Mycobacteria

## Abstract

Th17 cells are a recently discovered subset of T helper cells characterised by the release of IL-17, and are thought to be important for mobilization of immune responses against microbial pathogens, but which also contribute to the development of autoimmune diseases. The identification of C-type lectin receptors which are capable of regulating the balance between Th1 and Th17 responses has been of particular recent interest, which they control, in part, though the release of Th17 inducing cytokines. Many of these receptors recognise fungi, and other pathogens, and play key roles in driving the development of protective anti-microbial immunity. Here we will review the C-type lectins that have been linked to Th17 type responses and will briefly examine the role of Th17 responses in murine and human anti-fungal immunity.

## 1 Introduction

Effector T helper cells were originally classified as Th1 or Th2 cells, characterised largely by the production of IFNγ or IL-4, respectively. More recently, a third subset of effector cell has been identified, the Th17 cell, which are characterised by the production of IL-17. The induction of Th17 cell differentiation has been an area of considerable focus, and it is now appreciated that the development of these cells involves cytokines including TGF-β, IL-1β, IL-6, IL-21, and IL-23, and transcription factors including retinoic acid receptor-related orphan nuclear receptors (ROR) α and γt [1]. However, the actual involvement of cytokines, such as IL-23 and TGF-β, in the differentiation of Th17 cells has been controversial, as has the relatedness between human and mouse Th17 cells, although many of these controversies are starting to be resolved.

In addition to IL-17A and IL-17F, Th17 cells produce a variety of other cytokines, such as IL-21 and IL-22 for example, all of which are capable of inducing a variety of inflammatory and anti-microbial responses in other cell types, including myeloid and epithelial cells [1]. Consequently, Th17 responses have been implicated in driving protective immune responses against a variety of microbes, including several bacterial and fungal pathogens [2]. However, the inflammatory effects of these responses have also been implicated in the development of autoimmune diseases, such as experimental autoimmune encephalomyelitis [1]. Thus the protective anti-microbial effects of Th17 immunity can also contribute to pathology, perhaps explaining some of the controversy surrounding the role of these responses in murine models of fungal infection (discussed later).

Another area of interest has been the identification of the pattern recognition receptors (PRRs) and intracellular signalling pathways in antigen presenting cells (APCs) which drive the development of Th17 immunity. One group of PRRs which appear to have an emerging role in these types of responses are the C-type lectin receptors (CLRs), and particularly those that signal via the Syk/CARD9 pathway. As we shall see, many of these receptors are involved in fungal and bacterial recognition, and are capable of directly stimulating the development of adaptive immune responses. Thus these receptors act as archetypical PRRs, with functions analogous to those of the Toll-like receptor (TLR) family. In this review, we will examine each of the CLRs which have been implicated in Th17 immunity, and we will also briefly discuss what is known about the role of this type of adaptive response in anti-fungal immunity.

## 2 C-type lectins and Th17 responses

**Fig. 1:**
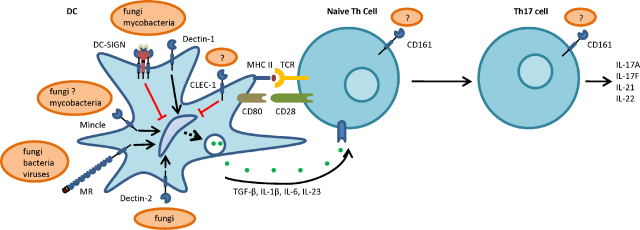
Cartoon representation of the roles of CLRs during the development of Th17 responses.

The C-type lectin-like receptors (CLRs) are a diverse family of proteins that are characterised by the presence of one or more C-type lectin-like domains (CTLDs). The CTLD is normally involved in ligand recognition and consists of a distinct protein fold that is created by disulphide bridges between conserved cysteine residues [3]. The CLRs are found as both soluble and membrane bound proteins and, although originally defined as carbohydrate binding proteins, are now known to be able to recognise a wide variety of other exogenous pathogen associated molecular patterns (PAMPs) and endogenous ligands, including proteins and lipids. Some of the membrane bound receptors are capable of triggering intracellular signalling; either directly through integral cytoplasmic signalling motifs, or indirectly, through association with signalling adaptor molecules, such as the Fcγ chain (see Ref. [4] for a review). The intracellular signalling pathways induced by these receptors can either inhibit cellular function (which normally occurs with receptors containing immuno-receptor tyrosine-based inhibitory motifs or ITIMs), or these receptors can trigger cellular activation (through immuno-receptor tyrosine-based activation motifs or ITAMs), resulting in the stimulation of various cellular responses, including the induction of gene expression. Based on their phylogeny and domain organization, CLRs have been divided into 17 families [5], but of interest are members of the group II, V and VI receptors which have been implicated in Th17-type responses including Dectin-1, Dectin-2, Mincle, DC-SIGN, CLEC-1, and CD161 (a summary of the roles and ligands of these various receptors is shown in Fig. 1 and Table 1). Most of these receptors have also been implicated in anti-microbial immunity.

### 2.1 Dectin-1 (Clec7a)

Dectin-1 is a type II transmembrane protein that is expressed predominantly by myeloid cells (monocytes/macrophages, dendritic cells and neutrophils), although the receptor is expressed on some other cell types, including various populations of lymphocytes [6]. The highest levels of Dectin-1 expression are on inflammatory cells and on cells at portals of pathogen entry, and the levels of this receptor can be significantly modulated by cellular maturation, cytokines and other biological response modifiers [6]. Notably, expression of Dectin-1 can be induced on other cell types, including mucosal epithelium [7].

**Fig. 2:**
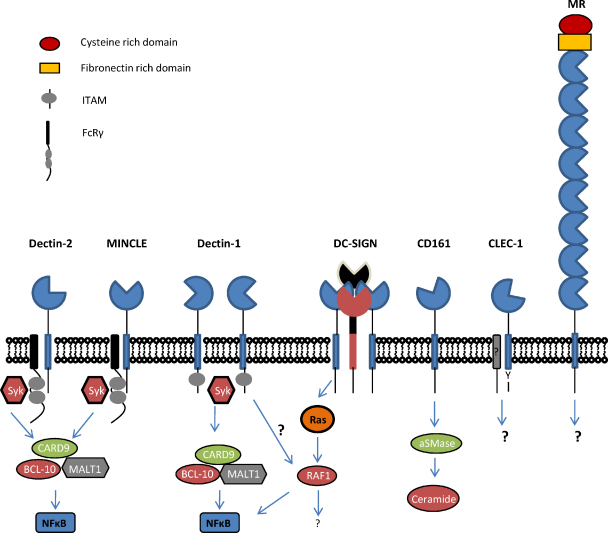
Cartoon representation of the structures and known signalling pathways that are employed by the various CLRs involved in Th17 responses.

Dectin-1 consists of a single extracellular CTLD, linked by a stalk and transmembrane region to a cytoplasmic tail containing an ITAM-like motif (also termed a hemITAM; Fig. 2) [6]. The receptor exists as two major isoforms, generated by alternative splicing, that differ by the inclusion of the extracellular stalk region and which possess slight functional differences [6]. Dectin-1 specifically recognises β1,3-linked glucans, carbohydrates found in the cell walls of fungi, and in plants and some bacteria. Dectin-1 recognises several fungal species, including a number of human pathogens such as *Candida*, *Aspergillus*, *Pneumocystis* and *Coccidioides*, and plays an important role in host defence against these pathogens [6,8,9]. Dectin-1 can also recognise mycobacteria and an endogenous molecule on T-cells, but the identity of the ligand(s) involved is unknown [6].

Ligand binding to Dectin-1 can induce a variety of cellular responses including ligand uptake by endocytosis and phagocytosis, cellular maturation, the respiratory burst, the production of arachadonic acid metabolites, and the production of numerous cytokines and chemokines, including IL-2, IL-10, CXCL2, TNF, IL-1β, IL-6 and IL-23 [6]. Dectin-1 can also interact with MyD88-coupled Toll-like receptors to synergistically induce the production of cytokines, including IL-6 and IL-23, but these interactions also result in the down regulation of IL-12 which is likely to contribute to the development of Th17 responses (see below) [10,11]. Signalling from Dectin-1 is mediated by the cytoplasmic ITAM-like motif and involves several downstream pathways, including those mediated through Syk/CARD9 and Raf-1 kinase [6].

Dectin-1 is also able to direct the development of adaptive immunity. Stimulation of Dectin-1 on antigen presenting cells (APCs) using highly purified β-glucans has been shown to induce the differentiation of Th17 and Th1 CD4^+^ T-cells and drive the development of antibody and CD8^+^ T-cell responses [12–14]. How Dectin-1 actually drives the development of Th17 responses is still unclear, although it requires both the Syk/CARD9 and the Raf-1 kinase signalling pathways, phospholipase C-γ2, and is likely to stem from the ability of Dectin-1 to stimulate (IL-1β, IL-6 and IL-23) and inhibit (IL-12) the production of cytokines important in shaping the development of these responses [11,14,15]. Stimulation of Dectin-1 on APCs, and the generation of IL-23, has also been shown to be capable of driving the conversion of selected populations of T_reg_ cells into IL-17 producing T-cells [16]. More recently, the direct stimulation of Dectin-1 on γδ T-cells, which are thought to contribute to early innate responses, induced the production of IL-17 from these cells; a response which could be substantially enhanced in the presence of IL-23 [17].

Several studies have suggested a role for Dectin-1 in driving IL-17 responses during fungal infection. In humans, a polymorphism of Dectin-1 has been identified which introduces a premature stop codon (Tyr238X) that prevents the expression of this receptor at the cell surface [9]. Individuals homozygous for this polymorphism are susceptible to mucocutaneous fungal infections and have defective production of cytokines, including IL-17, in response to fungi [9]. More recently, this polymorphism, and its effects on IL-17 production, have been associated with susceptibility to invasive Aspergillosis in stem cell transplant patients [18], and it was also associated with a decreased incidence of graft versus host disease that was linked with *Candida* colonization in these patients [19]. Interestingly, in murine models, Dectin-1 does not appear to play a major role in driving Th17 immunity in response to *C. albicans* [12], however this receptor was involved in generating protective IL-17 responses during experimental pulmonary infection with *A. fumigatus* [8].

In addition to fungi, Dectin-1 has been implicated in the development of adaptive responses to *Mycobacterium tuberculosis*. Several studies have suggested that Dectin-1 is involved in mycobacterial uptake and the induction of cytokines, such as IL-12, IL-23, IL-1β and IL-6, in response to these organisms [6,20]. Furthermore, recognition of mycobacteria by Dectin-1 on human APC *in vitro* was shown to promote the generation of Th1 and Th17 CD4^+^ lymphocytes, and the receptor may also be involved in the mycobacterial-mediated stimulation of IL-17 from γδ T-cells [17,20,21].

### 2.2 Dectin-2 (Clec-4n)

Dectin-2 is a type II transmembrane receptor expressed predominantly on tissue macrophages, DCs, and inflammatory monocytes [22]. The receptor possess a classical sugar-binding CTLD which recognises high mannose structures in a Ca^2+^ dependent manner, through which it recognises a variety of pathogens including capsule-deficient *Cryptococcus neoformans*, *Candida albicans*, *Saccharomyces cerevisiae*, *M. tuberculosis*, *Microsporum audounii*, *Trichophyton rubrum*, *Paracoccidioides brasiliensis* and *Histoplasma capsulatum* [23,24]. Dectin-2 also recognises allergens from house dust mites and fungi, and plays a role in UV-induced tolerance through the recognition of an unidentified endogenous ligand on CD4^+^ CD25^+^ T-cells [25,26]. Although the receptor has a short cytoplasmic tail which lacks traditional signalling motifs, Dectin-2 associates with the ITAM-containing FcRγ adaptor and can trigger intracellular signalling through the Syk-CARD9 pathway to induce a variety of cellular responses, including the production of ecosanoids, cytokines and chemokines (Fig. 2) [24,26–30].

Two recent studies, focussed on characterising the role of Dectin-2 in the host response to *C. albicans*, have provided the first evidence of a role for this receptor in the induction of Th17 responses. In these studies, Dectin-2 was shown to contribute to the induction of cytokines, including TNF, IL-6, IL-1β and IL-23, following stimulation of DC with fungal particles. Importantly, Dectin-2 signalling through the Syk/CARD9 pathway was shown to play a substantial role in the development of Th17 and Th1 responses upon *C. albicans* infection in mice, although the latter response also required Dectin-1 [28,29]. Using gene-deficient mice, Saijo *et al*. were additionally able to show that Dectin-2, which recognises fungal α-mannans, was essential for resistance to infection with *C. albicans* [28]. Based on these results, Robinson *et al*. have proposed that Dectin-2 and Dectin-1 account for nearly all of the Syk/CARD9-dependent signalling induced in response to these fungal pathogens [29].

### 2.3 Mannose receptor

The macrophage mannose receptor (MR; CD206) is a type I transmembrane protein which possess eight extracellular CTLDs and a short cytoplasmic tail which lacks classical signalling motifs (Fig. 2). Although the majority of the MR is located intracellularly, within the endocytic pathway, a soluble form of the receptor is also shed into the serum. The MR is expressed by macrophages, some DC, as well as a variety of other cells and tissues, including hepatic and lymphatic endothelia [31,32]. The MR can bind terminal mannose, fucose or N-acetyl glucosamine and consequently recognises a wide variety of endogenous and exogenous ligands, including several bacterial, viral and fungal pathogens (such as *C. neoformans*, *C. albicans* and *Pneumocystis carinii*) [32].

The MR has been shown to induce a variety of cellular responses, but the molecular mechanisms responsible for transducing the intracellular signals from this receptor are unclear. The recognition of fungi by this receptor, which may only occur in the phagosome following fungal uptake, has been shown to promote the production of a number of cytokines, such TNF, GM-CSF, IL-12, IL-8, IL-6, and IL-1β, although there is also evidence that the MR can inhibit the production of certain cytokines, including TNF [32–34]. Although the MR plays a clear role in homeostasis, its role in anti-microbial immunity is still unclear with MR deficient mice not showing significant alterations in immunity to *C. albicans* or *P. carinii* [35–37]. However these animals were found to be more susceptible to infection with *C. neoformans*, which resulted from defective induction of protective CD4^+^ T-cell responses [38].

Using *in vitro* assays with human PBMCs, Netea *et al*. recently demonstrated that stimulation of the MR with *Candida* or purified *Candida* mannan was able to induce significant levels of IL-17 [39]. This response was not a direct mitogenic stimulation of T-cells, as it required APCs, and both Dectin-1 and TLR2 were able to amplify these responses. This response was specific for *Candida*, as mannan isolated from *S. cerevisiae* had no effect [39]. In contrast to these results, however, another study demonstrated that stimulation of the MR could suppress Th17 responses induced by mycobacteria [20]. Understanding the signalling mechanisms utilised by the MR, and the effects of collaboration with different PRRs, are clearly issues that need to be addressed if the role of this receptor is to be fully understood.

### 2.4 Mincle (Clec4e)

Macrophage inducible C-type lectin (Mincle) is a type II transmembrane protein that is primarily expressed by activated macrophages, and probably also by DCs [40,41]. Like Dectin-2, Mincle possesses a single extracellular CTLD, a short cytoplasmic tail, and associates with the adaptor FcRγ to trigger intracellular signalling through the Syk/CARD9 pathway [42] (Fig. 2). Mincle recognises a variety of endogenous and exogenous ligands, such as necrotic cells, mycobacteria and certain fungi, including *Candida*, *Saccharomyces* and *Malassezia* (although the receptor may preferentially recognise the latter fungal species) [42–44]. Many of the ligands involved in these interactions have been identified and include fungal α-mannan, mycobacterial cord factor (trehalose-dimycolate as well as the synthetic analogue trehalose-dibehenate), and the small nuclear ribonucleoprotein SAP130 [42,44–46]. Upon recognition of these ligands, Mincle has been shown to induce a variety of cellular responses, including the induction of cytokines such as TNF, MIP-2, KC, IL-10 and IL-6. Mincle knockout mice show increased susceptibility to infections with *Candida*, and blocking Mincle function with antibodies *in vivo* has been found to reduce neutrophil recruitment and inflammatory cytokine production in response to necrotic cell death [42,43].

Of relevance here is the ability of Mincle to mediate immune responses to trehalose-dimycolate (TDM) and trehalose-dibehenate (TDB). TDM, which has potent inflammatory activity and is thought to be a key driver of pathogenesis during tuberculosis, and its less toxic analogue TDB, have been shown to be useful adjuvants for mycobacterial subunit vaccines in driving the development of protective Th1 and Th17 responses [41,47]. Recent data has shown that Mincle mediates all of the responses to TDM and TDB, with mice deficient in Mincle (or FcRγ chain) losing the ability to induce Th1 and Th17 responses following TDB/antigen immunization [45,46]. Thus like the other Syk-coupled C-type lectins described above, Mincle is able to direct the development of Th17 responses, and although only shown so far for mycobacteria, this receptor is likely to also contribute to the development of these responses during fungal infection.

### 2.5 DC-SIGN

Human DC-SIGN (CD209) is a type II transmembrane protein possessing a single extracellular CTLD, and a cytoplasmic tail containing internalization motifs (Fig. 2). DC-SIGN is expressed as a tetramer, due to interactions between the extracellular stalk regions of the monomers, and the receptor is expressed primarily by immature DC, but it is also found on selected macrophages and endothelial cells [48–50]. Mice express eight orthologs of this receptor, which differ slightly in their structure and expression profiles [51,52]. DC-SIGN recognises carbohydrates, including high-mannose and fucosylated structures, enabling it to recognise a wide variety of pathogens including mycobacteria and several fungal pathogens, such as *Candida* species, conidia of *A. fumigatus*, *Chrysosporium tropicum*, and possibly also *C. neoformans* [51,53–55].

Although the exact mechanisms are still unclear, DC-SIGN is able to induce intracellular signalling resulting in cellular responses, such as phagocytosis [55]. DC-SIGN signalling is mediated, in part, through the Raf-1 kinase pathway, and can modulate cytokine production induced through other PRRs, including the TLRs, although the receptor does not appear to be able to directly induce the production of cytokines [56]. The signalling induced by DC-SIGN depends on the nature of the carbohydrate ligand, resulting in the stimulation or repression of a number of cytokines including IL-10, IL-12 and IL-6 [56,57]. Indeed, a recent study has demonstrated that stimulation of DC-SIGN using defined ligands could inhibit the production of cytokines, including IL-1β, IL-23 and TNF, that were induced by *M. tuberculosis* or beta-glucan in human DCs, and that this led to a repression of Th17 responses [20]. Thus, although only demonstrated in one study to date, these results suggest that signalling from DC-SIGN can modulate the development of Th17 responses.

### 2.6 CLEC-1 (CLEC-1A)

CLEC-1 is a relatively poorly characterised member of the Dectin-1 cluster of C-type lectins, and is expressed by endothelial cells, DC and other tissue myeloid cells, but not by monocytes, granulocytes, B, T or NK cells in peripheral blood [58,59]. This type II transmembrane receptor is structurally similar to Dectin-1, although it does not contain any canonical signalling motifs, and may associate with an adaptor for expression at the cell surface [58]. However, the cytoplasmic tail does possess a single tyrosine residue, which may be able to induce intracellular signalling (Fig. 2). Expression of CLEC-1 was found to be down-regulated by inflammatory stimuli, such as LPS or IFNγ, but upregulated by immunosuppressive cytokines, such as IL-10 and TGF-β [60]. Interestingly, in rodent models, CLEC-1 was found to be upregulated in tolerated allografts, and this was associated with low levels of IL-17 and the presence of CD4^+^CD25^+^ T_reg_ cells at the graft site. *In vitro* assays demonstrated that CLEC-1 expression was increased on endothelial cells upon contact with CD4^+^CD25^+^ T_reg_ cells, and that loss of CLEC-1 expression from DC (using siRNA) enhanced the development of Th17 cells, while reducing Foxp3 expression, in MLRs [60]. Overall these results suggest that CLEC-1 plays a role in regulating the development of Th17 cells, but more data is need to fully understand the role and underlying mechanisms utilised by this receptor.

### 2.7 CD161

CD161 (NKRP1a) is a type II transmembrane receptor that consists of a single extracellular CTLD and a cytoplasmic tail that contains an atypical ITIM-like motif [61] (Fig. 2). The receptor is expressed by subsets of NK, NKT and T-cells, and has also been detected on monocytes and DC [61–63]. CD161 has been shown to recognise a number of ligands including carbohydrates, lectin-like transcript-1 (LLT1, CLEC2D), and proliferation-induced lymphocyte-associated receptor (PILAR) [64–66]. The role of CD161 is still unclear, but it has been shown to be capable of regulating NK cell cytolysis, trans-endothelial migration, and the proliferation of immature thymocytes, T-cells and NKT cells [62,64,65,67,68]. The receptor can also modulate the production of cytokines, including TNF, IL-1β, IFNγ, IL-4, IL-12, in various CD161^+^ myeloid and lymphoid cells [62,69]. How CD161 mediates these effects is still largely unknown, although its effects on T-cell proliferation were shown to involve interactions with PILAR, and signalling in NK cells has been shown to involve activation of acid sphingomyelinase [66,70].

Of relevance here is the use of CD161 as a marker of IL-17 producing human T-cells. Originally demonstrated to define subsets of CD4^+^ and CD8^+^ human T-cells with different functional activities, CD161 was subsequently shown to be highly upregulated in IL-17 producing T-cells, and in the precursor cells found in the umbilical cord and newborn thymus [71,72]. This led to the proposal that CD161 was a specific marker for Th17 cells, and subsequent studies have shown that this receptor is indeed expressed on all human IL-17 producing T-cell subsets, including those implicated in intestinal inflammation and in chronic inflammation induced by viral infection [72–75]. Interestingly, the mouse homologue (NK1.1) has not been found on IL-17 producing T-cells, which has contributed, at least in part, to the speculation that murine Th17 cells are different to those found in humans [76].

Despite the use of this receptor as a Th17 T-cell marker, its function on these cells is still unclear. Stimulation of this receptor on Th17 cells using antibodies had no effect on proliferation or cytokine production [72]. Given that Th17 cells can target specific tissues through the expression of chemokine receptors, such as CCR6, Cosmi *et al*. have proposed that CD161 may play a role in the trans-endothelial migration of these cells [72–74]. Clearly there is still much to be learned about the function of CD161 and its involvement in these responses.

## 3 Th17 in anti-fungal defence

Protective immunity to most fungal infections requires adaptive immune responses involving CD4^+^ T-cells, as evidenced by the susceptibility of HIV-patients to infections with fungal pathogens such as *Candida* and *Cryptococcus*. Historically, protection from fungal infections was thought to require Th1 immunity, while Th2 responses promoted susceptibility [77]. More recently, Th17 responses have also been implicated in anti-fungal immunity and, as we discuss below, data from both human and mouse have suggested that these responses are required for protection [78,79]. However, the protection mediated by these responses may be limited to particular sites (such as the oral mucosa) and there is evidence that they may also have detrimental effects during infection.

### 3.1 Human Th17-related fungal diseases

In the last few years several genetic causes for alterations in Th17 immunity have been identified which are linked with defects in anti-fungal immunity, including mutations in STAT3 (Hyper IgE syndrome; HIES), AIRE (autoimmune polyendrocrinopathy with candidiasis and ectodermal dystrophy; APECED), Dectin-1 (which was discussed above) and CARD9 [80]. There are also reports of patients with other mutations, such as those resulting in deficiency in IL-12p40 and IL-12Rβ1, which result in reduced levels of Th17 cells and increased susceptibility to fungal infections, but these are less well characterised [80,81]. In all cases, most affected individuals suffer from chronic mucocutaneous candidiasis (CMC); a heterogenous group of diseases in which patients suffer from persistent or recurrent *Candida* infections of the mucous membranes, skin and nails (onchomycosis).

#### 3.1.1 Hyper IgE syndrome (HIES)

Autosomal dominant-HIES, or Job's syndrome, is a complex immunodeficiency characterised by high serum IgE levels, atopic dermatitis, susceptibility to staphylococcal and fungal infections, and a variety of other non-immunological disorders, including bone and dental abnormalities. More than 80% of these patients suffer from CMC, but infections with other fungi, including *Aspergillus*, *Cryptococcus* and *Pneumocystis* have also been reported [82]. The major underlying defect was identified as a loss of function of signal transducer and activator of transcription 3 (STAT3), a result of dominant negative mutations in various regions of the protein [83,84]. STAT3 is activated following stimulation with several key cytokines involved in Th17 differentiation, including IL-6, IL-21 and IL-23, and it is required for the induction of RORγt, downregulation of FoxP3 in T_reg_ cells, and expression of IL-23R and IL-17 itself [85]. Several studies have demonstrated that T-cells from patients suffering from HIES did not produce IL-17 and were unable to differentiate into Th17 cells (for examples see Refs. [81,86,87]).

#### 3.1.2 Autoimmune polyendrocrinopathy with candidiasis and ectodermal dystrophy (APECED)

APECED, also known as autoimmune polyendocrine type I syndrome (APS-1), is a rare autosomal recessive disease characterised by an autoimmune-mediated destruction of various endocrine glands, resulting particularly in hypoparathyroidism and adrenal insufficiency, and a host of other abnormalities, including ectodermal dystrophies [88]. Another characteristic of the disease, and often one of the first indicators, is CMC. The majority of patients with this disease (>95%) have been found to have mutations in the autoimmune regulator (AIRE) gene which render it non-functional [89,90]. AIRE is involved in central tolerance, by controlling the production of self-antigens in the thymus and peripheral lymphoid organs, and loss of function of this regulator gives rise to the development of autoimmunity [88]. Two recent studies have demonstrated that these patients make neutralizing autoantibodies to Th17 cytokines, including IL-17A, IL-17F and IL-22, and the loss of these cytokines correlates with the increased susceptibility to CMC [91,92]. The production of similar autoantibodies was also found to correlate with the rare occurrences of CMC infection that are observed in certain thymoma patients [91].

#### 3.1.3 CARD9 deficiency

CARD9 (caspase recruitment domain-containing protein 9) is a key signalling adaptor in myeloid cells, that forms a complex with B-cell lymphoma 10 (BCL10) and mucosa-associated lymphoid tissue (MALT1) to transduce signals that activate mitogen-activated protein kinases (MAPK) and nuclear factor (NF-κB) [93]. CARD9 mediates the signalling downstream of Syk kinase, triggered from the ITAM-coupled receptors including Mincle, Dectin-2 and Dectin-1 (discussed above; see Fig. 2), leading ultimately to the production of numerous cytokines and chemokines and the induction of Th17 responses [12]. In mice, CARD9 has been shown to play an essential role in anti-fungal and anti-bacterial immunity [93–95]. In humans, a CARD9 mutation has recently been identified which gives rise to a premature stop codon (Q295X) [96]. Individuals homozygous for this mutation suffer from CMC and other dematophyte infections, and a number of patients also had invasive candidiasis (some of which died from these infections). Correlating with defective signalling through the CARD9 pathway, the affected individuals had a much lower proportion of Th17 cells than healthy controls, and *in vitro* experiments demonstrated that cells possessing this mutation were significantly impaired in their ability to induce inflammatory responses to fungal particles [96].

### 3.2 Mouse models

The human studies discussed above strongly suggest that Th17 responses are protective, but the data to date are only correlative. However, data from studies using knockout mouse models are not much better, and the role of Th17 responses in anti-fungal murine immunity (mostly with *C. albicans*) is still somewhat controversial. On the one hand, Th17 responses have been shown to confer protection in both systemic and oral mucosal models, which has been ascribed to the beneficial effects of IL-17 on the induction of anti-microbial effectors and neutrophil recruitment [78,97,98]. Similar protective effects of Th17 responses have also been shown for *Cryptococcus neoformans* and *P. carinii* [99,100]. On the other hand, different groups have reported that these responses either do not play a role (systemic infection), or are actually detrimental to the host (gastric mucosal and systemic infections), as a result of inappropriate IL-17-mediated neutrophil activation and resultant tissue pathology [101,102]. There are also similar contradictory data on the role of IL-22 [98,103]. The reasons for these differences are still unclear, but may be related to the choice of model (e.g.: oral versus gastric infection, which may suggest tissue specific protective effects of Th17 responses), mouse background (e.g.: Balb/C or C57BL/6) or knockout tested (e.g. IL-17A, IL-17R, etc.). These differences may also be a result of diverse fungal strains being used, which are recognised by different PRRs and which can also actively suppress Th17 responses [104,105].

## 4 Conclusions

Since the identification of Th17 cells much progress has been made in understanding the role and mechanisms involved in establishing this type of adaptive immunity. The C-type lectins have been of particular interest, as they have been implicated in inducing and modulating these types of responses. However, as highlighted during this review, there is still much we do not know and many controversies still to resolve, particularly with the mouse models of fungal infection. Gaining a better understanding of the functions of these C-type lectins, and their involvement in Th17 responses, should aid in the future development of novel vaccination strategies and may suggest avenues to enhance protective immune responses to infection.

## Figures and Tables

**Table 1:** C-type lectin receptors (CLRs), their ligands, and their role in the development of Th17 responses.

CLR	Ligands	Pathogens recognised	Role in Th17 cell responses
Dectin-1	*Exogenous*: β1,3-glucan	*Fungi*: *Candida*, *Aspergillus*, *Pneumocystis*, *Coccidioides*, *Saccharomyces*	Promotes Th17 responses to fungi and mycobacteria
	*Endogenous*: T-cell ligand?	*Bacteria*: mycobacteria	

Dectin-2	*Exogenous*: High mannose structures	*Fungi*: *Cryptococcus*, *Candida*, *Saccharomyces*, Microsporum, Trichophyton, Paracoccidioides, Histoplasma	Promotes Th17 responses to fungi
	*Endogenous*: T-cell ligand?	*Bacteria*: mycobacteria	

Mannose receptor	*Exogenous*: terminal mannose, fucose, N-acetyl glucosamine, sulphated sugars	*Fungi*: *Candida*, *Cryptococcus*, *Pneumocystis*	Unclear: suppresses Th17 responses to mycobacteria, but induces these responses to *Candida*.
	*Endogenous*: Various e.g. glycoprotein receptors, hormones, lysosomal enzymes	*Bacteria*: mycobacteria, Klebsiella, Streptococcus	
		*Virus*: Dengue, HIV	
		*Protozoa*: Leishmania	

Mincle	*Exogenous*: α-mannan, mycobacterial cord factor	*Fungi*: *Candida*, *Saccharomyces*, *Malassezia*	Promotes Th17 responses to mycobacteria (probably also fungi)
	*Endogenous*: SAP130		

DC-SIGN	*Exogenous*: high mannose and fucosylated structures.	*Fungi*: *Candida*, Chrysosporium, *Aspergillus*	Unclear: but may suppress Th17 responses.
	*Endogenous*: Various e.g. intercellular adhesion molecule (ICAM)-2 and 3, Lewis-x	*Bacteria*: mycobacteria	
		*Virus*: HIV	

CLEC-1	*Exogenous*: ?	?	Unclear: but may suppress Th17 responses?
	*Endogenous*: T-cell ligand?		

CD161	*Exogenous*: ?	?	Th17 marker
	*Endogenous*: PILAR, CLEC2D		T-cell proliferation?
			Cell migration?
			Regulation of cytokine production?
